# Endothelial cells and organ function: applications and implications of understanding unique and reciprocal remodelling

**DOI:** 10.1111/febs.15143

**Published:** 2019-12-04

**Authors:** Moritz Reiterer, Cristina M. Branco

**Affiliations:** ^1^ Centre for Cancer Research and Cell Biology Queen’s University Belfast UK; ^2^ Department of Physiology Development and Neuroscience University of Cambridge UK

**Keywords:** endothelial cell, metabolic reprogramming, microvasculature, organ‐specific, tissue remodelling

## Abstract

The microvasculature is a heterogeneous, dynamic and versatile component of the systemic circulation, with a unique ability to locally self‐regulate and to respond to organ demand and environmental stimuli. Endothelial cells from different organs display considerable variation, but it is currently unclear to what extent functional properties of organ‐specific endothelial cells are intrinsic, acquired and/or reprogrammable. Vascular function is a fundamental pillar of homeostasis, and dysfunction results in systemic consequences for the organism. Additionally, vascular failure can occur downstream of organ disease or environmental stress, often driving an exacerbation of symptoms and pathologies originally independent of the local circulation. The understanding of the molecular mechanisms underlying endothelial physiology and metabolism holds the promise to inform and improve diagnosis, prognosis and treatment options for a myriad of conditions as unrelated as cancer, neurodegeneration or pulmonary hypertension, and likely everything in between, if we consider that also treatments for such conditions are primarily distributed via the bloodstream. However, studying endothelial function has its challenges: the origin, isolation, culture conditions and preconditioning stimuli make this an extremely variable cell type to study and difficult to source. Animal models exist but are neither trivial to generate, nor necessarily adequately translatable to human disease. In this article, we aim to illustrate the breadth of microvascular functions in different environments, highlighting current and pioneering studies that have advanced our insight into the importance of the integrity of this tissue, as well as the limitations posed by its heterogeneity and plasticity.

## Graphical abstract

The endothelial cell (EC) layer is a continuous organ, which enables tissue function, regeneration and homeostasis. Along the vascular tree, EC assume the adequate morphology, adhesion, metabolism and signalling to perform those functions. This allows adequate supply to the resident microenvironment and adjustments to variations in nutrients, oxygen or pathological conditions. EC plasticity, versatility and heterogeneity can be better understood and exploited to understand organ function, disease and treatments.

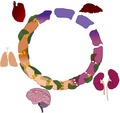

**Abbreviations**
EC: endothelial cells
NO: nitric oxide
NOS: nitric oxide synthase
NO_X_: nitric oxides
ROS: reactive oxygen species
VEGF: vascular endothelial growth factors

## Background

The microvascular system is arguably the largest organ in the human body, covering a surface area of 1–7 m^2^, and only rivalled in mass by the liver and skin 1. It is also likely the most heterogeneous 2. What can be in basic terms defined a continuous single cell layer of endothelial cells (EC), for the most part, is known for its importance as a vehicle for transport of substances, signals and other cells throughout the organism and between tissues. This essential function is at the heart of homeostasis 2, 3, as it facilitates the adjustments needed to meet the demands of individual tissue types in specific circumstances. This includes normal physiological conditions, such as during exercise, and exposure to altitude or temperature oscillations 4, or in pathologies such as inflammation 5, 6, cancer 7, 8, 9, ageing 10, degeneration 11, 12 or wound healing/tissue remodelling 13, 14, 15, 16. Another point for consideration is the EC response during systemic therapy for chronic or acute conditions 17, 18. Simply, this system keeps the organism connected and functioning as such.

The transport function of blood vessels is mostly centrally regulated. Blood flow is controlled autonomically through regulation of heart rate and peripheral resistance. However, within organs and tissues, local control of perfusion 10, 19, 20, 21, 22 in specialized capillary networks often bypasses autonomic control 20, 22, 23, 24 and the flow rate is adjusted to the tissue environment. Importantly, local adjustments in blood flow in specific organs, as a result of changes in EC activation, can result in systemic effects 19, 25. One could consider that local control in those instances indeed overtakes autonomic influence.

The delivery function of the microvasculature, or the movement across EC monolayers, is a complex process that, again in an organ‐specific manner, can occur in diverse forms, either para‐ or transcellularly, in a tightly regulated manner, as discussed and reviewed elsewhere 26, 27.

In the majority of healthy tissues, the endothelium is thought to be mostly quiescent and the movement of cells and compounds across this barrier results from a dynamic and integrated response of EC to compounds and cells in circulation, combined with local tissue microenvironment and metabolites at any given time.

The importance of local regulation of vascular functions is underscored by the heterogeneity of EC 2, 3, 24 in terms of morphology 28, 29, structure 30 and barrier function 2. The morphological and functional diversity along the vascular tree is in great part due to the endothelial glycocalyx, a dense grid of proteoglycans, glycosaminoglycans, glycoproteins and glycolipids, found on the luminal side of EC. The glycocalyx is present in vessels of all types and sizes, but organ‐specific differences are increasingly being discovered: in the sinusoidal capillaries of the liver, the glycocalyx is thin 31, whereas in the glomerular endothelium fenestrae, it provides an additional filtration barrier 32. Amongst continuous endothelia, the glycocalyx in the brain microvasculature is especially dense and resistant to lipopolysaccharide‐induced vascular injury 33; the authors speculate that this may contribute to blood–brain barrier function. Its size correlates to vessel diameter and ranges from several 100 nm in capillaries up to 10 μm in the carotid artery 34, 35, and thus, this structure directly affects organ perfusion.

Other microvascular properties that are unique to certain microenvironments include angiogenic potential 7, 19, 23, 24, 36, angiocrine/endocrine profile 20, 37, 38, 39 and metabolic rates 24, 36, 40, 41; all are essential functional parameters, and all are tissue‐specific.

Acknowledging this complexity, as well as the distinction between intrinsic EC properties and those that are programmed by their surrounding environment, is fundamental to understand and build on the potential for applications of (micro)vascular health in organ and organism performance.

A large body of work has been dedicated to EC in very unique pathologies, such as in the tumour vasculature 36, 41, 42 and diabetic retinopathy 13, 43, 44, 45, which have provided valuable breakthroughs in vascular biology and metabolism. Yet, this knowledge is limited in the representation of the impact of EC dysfunction as both cause and effect of other conditions, including lifestyle and ageing, which can neither be generalized and applied to all EC populations, nor seamlessly translated between model organisms.

This viewpoint article will not review detailed molecular aspects of current knowledge of EC biology; instead, it will emphasize general aspects of local control of vascular function and the challenges of adequately modelling these studies, while providing an overview of existing new and exciting developments in the field, to highlight the potential applications and implications of harnessing microvascular properties to improve human health.

## Regulation and regulators of EC function in different tissues

**Figure 1:**
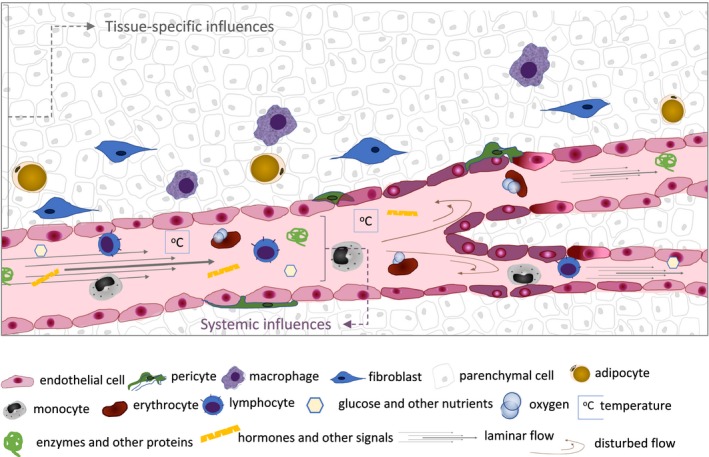
Overview of biological, chemical and physical influences on EC behaviour. Endothelial cells in capillary networks are exposed to tissue‐specific cues, which include signals from resident parenchymal and other stromal cells, the composition and stiffness of the ECM and the metabolic activity of the organ at any given time, which in turn affects the metabolite and gas composition, and extracellular pH. From the luminal side, EC perceive and respond to compounds and cells transported in circulation, such as nutrient status, circulating cells and oxygen levels (systemic influences); tissue‐specific influences include temperature and shear stress, which is altered as a function of vessel diameter, flow and branching; additionally, resident cells and metabolic, physiological and pathological status of specific organs in specific circumstances will provide the endothelium cues with very localized (tissue‐specific) relevance, but which can, too, provide systemic signals (e.g. angiocrine/endocrine). The activation status of the EC dictates its permeability, angiogenic potential, surface receptors and transporters, secretory profile and metabolism, and thus organ function.

Microvascular function is currently too loose a term to reflect the functional heterogeneity observed along the vascular tree, not only in terms of vessel type but also location within specialized tissues. Recent studies have underscored the unique and essential traits of EC, and how they are often the guardians of organ performance, as well as the initiators of tissue adaptation and regeneration; conversely, many pathologies are exacerbated as a result of effects on microcirculation or caused by inadequate vascular responses in the initial stages of disease. An overview of the range of stimuli affecting EC is illustrated in Fig. 1.

**Figure 2:**
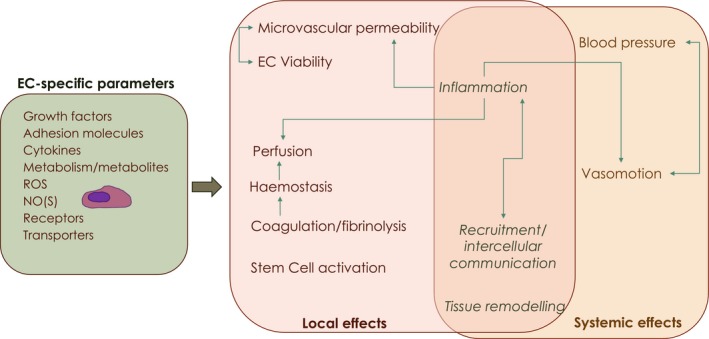
Summary of local and systemic effects of specific EC parameters. Endothelial cells possess diverse combinations in surface receptors and transporters, as well as diffusible secretory compounds, which include growth factors (such as VEGF) and metabolites, which will be determined by intrinsic endothelial properties and tissue microenvironment/substrate availability; receptors, transporters and signalling molecules such as reactive oxygen species (ROS) or nitric oxide (NO), downstream of endothelial or inducible nitric oxide synthases (NOS), are also variables contributing to EC heterogeneity and plasticity. All these parameters are specific to individual capillary networks, but oscillate, in a more or less transient fashion, in response to local and systemic pressures. These alterations in endothelial behaviour, signalling and metabolic activity subsequently modulate local tissue microenvironment as well as systemic circulation patterns.

Systemic stimuli are presented to EC before any other cells in the organism (e.g. circulatory factors, environmental oxygen, hormones), and though the downstream responses are unique to the tissue context, all EC are equipped to identify changes in essential circulating factors, such as oxygen or energetic nutrient load, as well as the mechanical stimulus of laminar flow. The combination of hormone receptors, adhesion molecules, junction type and density and secretory patterns allows and conditions each endothelial network response while protecting the tissue it supplies; these responses can result in organism‐wide effects in blood flow and distribution (as summarized in Fig. 2).

### Endothelial Hypoxia‐inducible Factors – beyond O_2_

Oxygen sensing is essential for homeostasis and management of cellular energy, and one of the most important roles of the vasculature. It has been extensively described in the lung endothelium 5, 14, 38, 46, which distinctly responds with vasoconstriction to drops in atmospheric oxygen, instead of the vasodilation observed in every other capillary network; The effect of O_2_ availability has also been thoroughly studied in tumours, where the environment is hypoxic, perfusion is deficient, and angiogenic factors are abundant 9, 41, 42. The sensing and response to atmospheric oxygen levels are vital to mammalian energetic strategy, and the skin is a known key peripheral sensor that can alone modulate systemic blood flow 47.

As in all mammalian cells, the master regulators of the hypoxia response in EC are the hypoxia‐inducible transcription factors (HIF). These factors coordinate transcriptional programs downstream of their activation, following stabilization of their regulatory subunits with the most important being HIF‐1α and HIF‐2α 48, 49, 50, 51. In EC, these are not only stress response factors, but act also, and primarily, as physiological regulators 14, 46, 52.

HIF‐2α is thought to be constitutively expressed in EC and essential for the maintenance of monolayer stability and barrier function 46, 53, through the regulation of vascular endothelial (VE)‐cadherin expression and localization at the cell surface. VE‐cadherin is the adhesion molecule found in endothelial adherens junctions and essential for reversible vascular permeability 54, 55. Furthermore, it is an important regulator of cytoskeleton structure and cell architecture, as well as intracellular signalling via initiation of transduction cascades, and it is thought to be determinant in the establishment of EC polarity 46, 56. Even though HIF‐2α in EC is vital for vascular integrity, stability and recovery 38, 46, 52, it is also required for the onset and exacerbation of pulmonary hypertension 14, 57, and deletion of lung EC HIF‐2α resulted in ameliorated hypertension phenotypes in mice 14. In general, HIF‐2α is seen as the gatekeeper of EC quiescence, but depending on the tissue or the combined parameters within certain conditions, stabilization of endothelial HIF‐2α is not advantageous.

HIF‐1α can be seen as the disruptor of EC quiescence. This isoform is more ubiquitously expressed in mammalian cells 58, and its role in EC function, like in most other cells, is usually of a transient nature, occurring as a result of demand, change or insult 59. HIF‐1α causes EC activation, which is a procoagulant 60, pro‐inflammatory 38, 61, 62 and pro‐angiogenic state 3, 51. This function is essential for new vessel formation to accompany growth and development, as well as for EC reshaping and migration, metabolic reprogramming 36, 41, 62 and recruitment of other cells to sites of injury and inflammation 38, 51, 61. Activated EC are prothrombotic 63, due to the surface expression of intercellular adhesion molecules that promote platelet interaction and binding of circulating myeloid cells 3, 64. Essential as this function is for control of blood flow and tissue remodelling, it needs to be reversible. As such, activation of the coagulation cascade also and concomitantly stimulates fibrinolytic function, in an intricately choreographed and critically timed series of events 3. Constitutively high levels of endothelial HIF‐1α are known to potentiate a number of pathologies, including hypertension 62, 65 and cancer, when HIF‐1α permanence as a result of hypoxia incurs a vicious cycle of angiogenesis and inflammation, with consequences in tumour cell migration and aggressiveness 2, 36.

Many HIF‐derived functions are implemented via accumulation of vascular endothelial growth factor (VEGF) and downstream receptors and effectors 28, 49, 51, 66, 67. The levels and source of VEGF, as well as what isoforms are present, dictate the behaviour of both EC and their relation to the surrounding tissue.

However essential the HIF‐regulated pathways are in adequate adaptation to oscillations in oxygen levels, it has become increasingly evident that their stabilization and activity in EC are graded and complementary, and occur in response to a myriad of other parameters, frequently independent of oxygen levels. HIF and VEGF signalling are also involved in the regulation and the responses to signals affecting EC function, including reactive oxygen and nitrogen species, cell proliferation, cell death and shear stress resulting from oscillations in laminar flow.

### ROS, NO_X_ and other regulators of EC behaviour

EC function is all but static and effective as a summation and processing of multiple stimuli by individual cells, often within close proximity along the same vessel segment. Signalling affecting EC behaviour often involves generating and responding to reactive oxygen species (ROS) 68, which can be generated by the EC themselves or by neighbouring cells, as a result of metabolic shifts, injury or damage, or an immune response 25, 68, 69, 70. It has been shown that the ROS‐associated antioxidant machinery expressed in EC is unique to vessel type and location within the vascular tree and organ of origin 69, suggesting that tolerance to oxidative stress is tissue‐specific, and some EC are more responsive/susceptible to oxidative damage than others.

Nitric oxide (NO) is intrinsically generated by EC primarily via eNOS 16. NO effectively modulates microvessel permeability and vasomotion by mediating changes in vessel calibre through relaxation of smooth muscle 19, 37, 71. However, this effect is, too, context‐specific: in adipose tissue of patients with metabolic syndrome, the hypoxia‐derived NO is an ineffective vasodilator 19, possibly because the endothelium senses hypoxia as a lesser threat than that of energetic nutrient overload, and bypasses that signal in favour of vasoconstriction instead, with hypertension as a systemic consequence. It has also been shown that damage to the EC glycocalyx abolishes subsequent changes in EC gene expression and NO production 72, 73.

EC are the only cells continuously exposed to laminar flow, to which vessel diameter and blood fluidity need to be adjusted in order to maintain perfusion. Their ability to endure and resist shear stress relies on ROS and NO_X_ signals, as well as epigenetic control 25, 30, 72, 73. Versatility and flexibility are essential EC properties, such that periods of altered cardiac output are not mirrored by altered organ function. Dysfunctional, disproportionate or irreversible responses result in conditions such as renal failure 74, exacerbation of COPD 5, 75 and hypertension 10, 61, 65. Neurodegenerative conditions and deficient muscle blood flow in the elderly can also occur or deteriorate downstream of failing NO signalling (and ROS scavenging) 11, 12, 37.

Additionally, specialized vascular beds are also regularly remodelled under hormonal control, such as mammary glands during pregnancy, lactation and involution 15, or the recurrent transformation of the uterine lining, downstream of cyclical periods of quiescence and angiogenesis 76. Recent studies in hUVEC have suggested EC can behave as peripheral circadian clocks, as cycles of cytokine expression and components of the coagulation cascade appear to be expressed in circadian patterns 77.

The collective function of a microvascular network requires adequate individual EC responses, but also appropriate and rigorous assembly. This involves the right cell shape and size, which directly affect vessel calibre and perfusion 29, 30, 72, alignment 62, 78 and, no less important, cell number. The proliferative stage of the angiogenic process is followed by a maturation phase, and EC number is therefore tightly controlled, by ongoing removal of accessory or interfering cells. Thus, EC apoptosis is essential to allow appropriate regression, to improve network functionality and to allow selective removal of superfluous EC during vessel maturation 15. It has also been shown to precede the apoptosis of myocytes to allow recovery from ischaemia/reperfusion injury, in a eNOS/VEGF‐dependent manner 79.

Recently, autophagy was revealed to be vital in microvascular health 80, identified as cytoprotective and fundamental for appropriate cell alignment 81; inefficient EC autophagy was shown to lead to EC senescence and lipid retention, inflammation and plaque burden, and thus fostering atherosclerotic lesions 78, 82.

Interestingly, and illustrating the importance of control of EC number, human pulmonary hypertension pathology includes EC accumulation in precapillary arterioles, a parameter that is not reproduced in rodent models 83.

### Metabolic shifts as mediators of EC form and function

In recent years, the interest in EC metabolism has grown. Pioneering studies in the unique tumour microenvironment demonstrated that metabolic switches are not mere bystanders but play active roles in shaping EC behaviours, such as angiogenic sprouting 84.

A crucial feature of EC metabolism is their reliance on aerobic glycolysis in favour of mitochondrial respiration. EC display markedly higher glycolytic rates than other cell types 85, while containing fewer mitochondria 86. Indeed, at least 75% of the ATP generated by porcine aortic EC derives from glycolysis 87. It has been postulated that this allows more oxygen to diffuse into tissues, while limiting the exposure of EC to potentially harmful reactive oxygen species 88. Furthermore, glucose supply to EC is generally abundant due to their direct exposure to blood. Therefore, glycolysis actually provides a faster means of energy production than oxidative phosphorylation. In conditions of increased or decreased glucose availability, EC can accordingly modulate the rate of mitochondrial respiration versus glycolysis 89, 90.

The glycolytic phenotype is exaggerated in tumour vasculature through upregulation of PFKFB3 (which mediates the conversion of fructose‐6‐phosphate to fructose‐2,6‐bisP) and glucose transporter 1 (GLUT1) 91, 92. Inhibition of these factors induces vessel normalization, thus reducing the risk of metastasis. PFKFB3 activity in this context is regulated by a wide variety of signals, including hypoxia, cytokines and hormonal stimuli 91, 93. Conversely, EC in established and mature vessels downregulate glycolysis in response to laminar flow 94.

Although these paradigms hold generally true, comparative studies between EC from different regions of the vascular tree, as well as between EC from different organs, have found substantial divergences in their metabolic phenotypes. For example, pulmonary venous EC rely more on aerobic glycolysis than their arterial counterparts *in vitro* 95. Heart microvascular EC show higher metabolic rates than those found in lung, liver and kidney, both in glycolysis and in mitochondrial respiration 24, whereas brain EC have a higher mitochondrial volume than other EC 96. In all cases, there has been little effort to investigate the underlying reasons or functional consequences of these differences *in vivo*, although it has been shown that mitochondrial inhibitors increase the permeability of the blood–brain barrier 97.

It is tempting to speculate that these various metabolic phenotypes represent an adaptation of EC to their individual niche, reflecting the specific needs of the underlying organ. For example, brain microvascular EC form part of the blood–brain barrier, transport across which is highly regulated through a host of influx and efflux pumps. It has been hypothesized that the higher mitochondrial density in brain microvascular EC is required to supply sufficient energy to fuel these transporters 84. Furthermore, glucose is the main source of energy for the brain 98. Thus, increased reliance on oxidative phosphorylation over glycolysis within brain EC allows more glucose to pass through the blood–brain barrier. Similarly, heart microvascular EC express higher levels of fatty acid transporters than other vascular beds, to account for the metabolic needs of cardiomyocytes. This feature is mediated by the transcriptional regulator Meox2/Tcf15, uniquely expressed in cardiac EC and lost upon *in vitro* culture 99.

Such loss of organ‐specific features *in vitro* is observed amongst several different EC 100, 101, but the mechanisms behind this phenomenon remain largely unknown. It stands to reason that the cause is a combination of loss of signals from neighbouring cells in the tissue, as well as a change in the physical environment. Depending on the exact mode of culturing, this may involve loss of the three‐dimensional vessel structure, loss of shear stress, changes in nutrient availability and increased exposure to oxygen. Given the crucial role of EC in oxygen homeostasis, exposure to hyperoxia in particular may affect EC physiology. Indeed, our own observations in culturing microvascular EC at different oxygen levels show that this parameter alters both their baseline metabolism and their ability to adapt to hypoxia (Reiterer *et al*., in preparation). Importantly, oxygen content within blood is not constant but varies drastically from 13% to 14% in freshly oxygenated arterial blood 102, to < 5% in peripheral organs such as the brain 103. Therefore, while the 21% O_2_ present in ambient air indeed provide a hyperoxic environment to all EC, the magnitude of the hyperoxic insult is less severe for cells from naturally well‐oxygenated tissues. This introduces a confounding variable if cells from different tissues are compared under ambient oxygen levels. For example, brain microvascular EC cultured at 5% O_2_ display a higher respiratory capacity than their lung counterparts but this trend is reversed if the cells are cultured at 21% O_2_ (Reiterer *et al.*, in preparation).

## Endothelial dysfunction and comorbidity: diagnostic, prognostic and treatment opportunities?

Insight into organ‐specific microvascular function provides an opportunity to tackle a vast amount of pathologies. Indeed, it is fair to speculate that it may provide the prospect of diagnosing and preventing an equally vast amount.

We know liver sinusoid EC, alone and in combination with Kupffer cells, play roles in innate and adaptive immunity, in recruitment and activation of monocytes and CD4^+^ T cells, are involved in 75% LPS clearing during acute inflammatory insults and hepatic regeneration following severe resection. Similarly, liver and lung tissue regeneration, extracellular matrix remodelling 104 or muscle recovery from ischaemic damage, all rely on appropriate revascularization, which is a result of a meticulous molecular and intercellular manoeuvring, both those initiated by EC and those that EC must respond to, such as the signals sent from perivascular environment 9, 10, 105, 106. The potent restorative powers of microvascular EC include using tissue‐native language to communicate with resident cells 16, but also activation of neighbouring stem cells 23.

However, and likely more often, the vasculature is overwhelmingly challenged by other conditions. Secondary to the primary cause of the malaise, the microvascular reactions to pathological challenges often result in either severe comorbidity, such as in the case of respiratory disease (*e.g.* COPD and acute respiratory distress syndrome) 5, 46, 75 or sepsis 107, 108, where EC activation compounds primary symptoms through added oedema, permeability and positive feedback of inflammatory signals. Endothelial activation in response to disturbed flow underlies atherosclerosis, a condition also shown to result from damage to the endothelial glycocalyx 109, 110 **,** but also results in a feed‐forward loop that exacerbates the condition and fosters the establishment of further and wide‐ranging complications. The same is observed in metabolic syndrome and the inability to coordinate a response to local vasodilating factors with the need to limit nutrient absorption and transport to an already overwhelmed tissue 19, 108.

Similarly to signals, cells, gas and nutrients, treatments are also commonly delivered to diseased tissues via the bloodstream, and it is this same system that transports waste metabolites for processing and removal from the organism. It is thus not surprising that the inevitable exposure to, and often absorption of, those compounds result in changes in EC viability and behaviour, and this is seen more strikingly during extreme cytotoxic treatments for cancer 17, 111. A significant body of work 17, 111, including some of our preliminary studies, show that minimal exposure of human and murine EC to the lowest (physiological) levels of chemotherapeutic agents, even for as little as 15 min, results in extensive changes in activation state and viability, most of which are not reversed after 72 h (Eakin et al., in preparation). Also, acute intestinal toxicity following radiotherapy is significantly compounded by associated EC dysfunction 18.

### Discussion: challenges, implications and applications for increased knowledge of specific EC populations

Finding accurate and representative models for *in vivo* EC behaviour has proven to be a challenging task. Immortalized EC derived from a wide variety of vessels are commercially available. While they avoid issues relating to donor heterogeneity, their modifications necessarily make them a less accurate model. Thus, *in vitro* studies have increasingly moved towards using primary EC instead. The large majority of such studies use human umbilical vein EC (hUVEC). These cells are relatively easy to culture and allow for highly reproducible experiments. However, their widespread use may lead to skewed observations, due to the heterogeneity of EC from different vessel types or from different organs. Organ‐specific primary EC are also used, but they are laborious to obtain or expensive to purchase. Cells from human donors additionally suffer from issues relating to donor heterogeneity, since samples are usually obtained from a small number of individuals who may differ significantly in age, gender and physiological state. Furthermore, the behaviour of EC is highly influenced by culture conditions. Most conventional growth media contain nutrients and/or growth factors in nonphysiological concentrations, leading to altered proliferation rates, metabolism and permeability 112, 113, 114, 115. Similarly, EC *in vivo* are constantly exposed to shear stress from blood flow. Removing this stimulus has been shown to alter EC metabolism 62, 94, 116.

While *in vivo* readouts are available, they usually involve one of a small number of assays, such as measuring angiogenesis in the retina or the hindbrain. Animal experiments frequently use zebrafish as a model system, since their transparent nature enables complex *in vivo* microscopic analysis and real‐time visualization of cellular behaviour *in situ*. However, findings are not always translatable into humans 55, 117, 118, 119. Knockout studies in mice using Cre recombinase under endothelial‐specific promoters are possible, but global phenotypes are often difficult to interpret or may even obscure organ‐specific effects. A challenge for the expansion of this subject will be to generate and establish reliable *ex vivo* models to study the molecular aspects of microvascular function, as well as expand the *in vivo* options to further study the reciprocal contribution of the organ microenvironment and thus provide increased translational value.

Altogether, the above examples stress a wide range of conditions that can be managed and treated also as a vascular disease, and that opportunities in which preventing vascular dysfunction could improve treatment outcomes and reduce morbidity associated with the primary condition. The knowledge of specific properties of tissue‐specific EC features and responses will allow the development of therapeutic strategies that are suitably targeted to the unique condition.

## Conflicts of interest

The authors declare no conflict of interest.

## Author contributions

CM outlined the structure and content, prepared figures and legends, researched, reviewed and wrote the abstract, background (Background) and sections on tissue‐specific regulation (Regulation and regulators of EC function in different tissues), hypoxia (Endothelial Hypoxia Inducible Factors – beyond O_2_) and signalling (ROS, NO_X_ and other regulators of EC behaviour). MR researched, reviewed and wrote section on metabolic regulation and influence on EC biology (Metabolic shifts as mediators of EC form and function) and contributed with own unpublished data for the review and discussion on heterogeneity and modelling studies of microvasculature (Endothelial dysfunction and co‐morbidity: diagnostic, prognostic and treatment opportunities?). CM and MR wrote the discussion (Discussion: challenges, implications and applications for increased knowledge of specific EC populations) and proofread the manuscript.
